# The effect of behavioral therapy based counseling with anxious mothers on their infants' colic: a randomized controlled clinical trial

**DOI:** 10.1186/s12887-022-03683-7

**Published:** 2022-11-08

**Authors:** Reihaneh Montazeri, Shirin Hasanpour, Mojgan. Mirghafourvand, Manizheh Mostafa Gharehbaghi, Mohammad Mehdi Ghods Tehrani, Shiva Mohajjel Rezaei

**Affiliations:** 1 Student Research Committee, Department of Midwifery, Nursing and Midwifery Faculty, Tabriz University of Medical Sciences, Tabriz, Iran; 2 Women’s Reproductive Health Research Center, Tabriz University of Medical Sciences, Alzahra Hospital, Artesh Street, Tabriz, Iran; 3 Social determinants of Health Research Center, Department of Midwifery, Nursing and Midwifery Faculty, Tabriz University of Medical Sciences, Tabriz, Iran; 4 Department of Pediatrics and Neonatology, Pediatric Health Research Center, Tabriz University of Medical Sciences, Tabriz, Iran; 5 Department of Pediatrics and Neonatology, Shiraz University of Medical Sciences, Shiraz, Iran; 6 Department of Psychology, Tabriz Branch, Islamic Azad University, Tabriz, Iran

**Keywords:** Counseling, Behavioral Therapy, Anxiety, Infantile Colic, Attachment, Iran

## Abstract

**Background:**

Given the possible effect of maternal anxiety on the severity of colic pain in infants, this study aimed to investigate the effects of behavioral therapy counseling on infantile colic (primary outcome), maternal anxiety, and mother-infant attachment (secondary outcomes) in anxious mothers with colicky infants.

**Method:**

This randomized controlled clinical trial was conducted on 46 anxious mothers of 2–6-weeks-old exclusively breastfed colicky infants who had a score of 112 and above according to the Postpartum Specific Anxiety Scale (PSAS), reffered to the pediatric clinics of Al-Zahra, Taleghani and Children Hospitals of Tabriz, Iran. The participants were randomly assigned to the intervention (*n* = 23) and control (*n* = 23) groups using randomized block design. Mothers in the intervention group attended 8 systematic desensitization counseling sessions (2–3 sessions per week). Those in the control group received routine care. The researcher completed the Postpartum Specific Anxiety Scale (PSAS), Mother-Infant Attachment Questionnaire (MIAQ), and Infant Colic Scale (ICS) by interviewing the participants before and two weeks after the intervention.

**Results:**

There was no significant difference between the intervention and control groups in the socio-demographic profile of participants. After the intervention, the mean postpartum anxiety score of women in the intervention group was significantly lower than that of those in the control group (Mean Difference (MD) = 22.5, 95% Confidence Interval (CI) = 2.3 to 42.7; *p* = 0.029). The mean infant colic score of the infants of mothers in the intervention group was insignificantly lower than that of those in the control group (MD = -2.9, 95% CI = -8.3 to 2.4; *p* = 0.271). In addition, no significant difference was observed between the two groups in terms of their mean mother-infant attachment scores (MD = -0.04, 95% CI = -3.1 to 0.3; *p* = 0.976).

**Conclusion:**

Behavioral therapy counseling effectively reduced postpartum anxiety in women with colicky infants; however, this reduction did not lead to a significant decrease in the infants’ colic pain. Therefore, health care providers are recommended to use this counseling method in combination with other effective counseling approaches to promote mental health of these mothers.

**Trial Registration:**

IRCT Registration Number: IRCT20111219008459N14, registered on 08/10/2020. https://irct.ir/user/trial/45949/view

## Introduction

Colic is often the first challenge that parents and infants face. This problem greatly affects their lives [1, 2]. According to Wessel, infantile colic is defined as uncontrollable crying in infants less than three months old that lasts at least three hours a day, three days a week, and three weeks a month [3, 4]. Other common symptoms include experiencing abdominal contractions, drawing legs up towards the abdomen, difficulty in defecation, arching back, and kicking [1, 5]. According to Talachian et al.'s study on 321 infants in Tehran, 20.2% of them met the criteria for colic based on Wessel's law [6]. Globally, approximately one in five infants under three months of age suffers from colic [1], and only 5% of infants with colic are diagnosed with gastrointestinal disease [2].

Two hypotheses have been suggested about the cause of colic including “abnormal gastrointestinal movements and pain signals from the sensitive areas of the intestine,” and “insufficient amounts of lactobacilli and increased levels of coliform bacteria that increase intestinal gas formation [7]”. In addition, behavioral problems such as anxiety, family tensions, and inadequate parent-infant interactions have been noticed in the etiology of colic [7, 8].

The brain may affect intestinal microbes through the hypothalamic-pituitary axis and the autonomic nervous system [9, 10]. Studies suggest that intestinal microbes interact with the central nervous system (CNS) probably through neural, endocrine, and immune pathways, and consequently affect brain function and behavior, and play role in the regulation of anxiety, mood, cognition, and pain [11]. The high correlation between psychiatric symptoms and gastrointestinal disorders (*e.g.* irritable bowel syndrome and inflammatory bowel disorder) highlights the importance of this axis in the pathophysiology of colic [12].

High maternal anxiety is associated with increased levels of cortisol in breast milk, which reaches its highest concentration 2–12 weeks after delivery [13]. On the other hand, increased cortisol level in breast milk is associated with the severity of crying and anxious behaviors in infants [14]. According to Maartje et al*.*, increased stress and cortisol levels in the middle of the day are associated with several changes in gut microbes [15].

Studies indicate that mothers of colicky infants are more disturbed, preoccupied, exhausted, and inflexible than those with non-colicky infants [16]. They also have difficulty performing their daily activities, and get high depression, fatigue, frustration, anger, anxiety, and overprotection scores than other mothers [17, 18]. Postpartum psychological problems are very common, and depression and its symptoms are still the most discussed, while less attention is given to anxiety [19, 20]. According to a study in Australia, among 408 mothers, 16% had anxiety disorder and only 4% had postpartum depression, therefore, the postpartum period can accelerate the development of anxiety symptoms [21]. High maternal anxiety is often associated with decreased self-efficacy [22], poor parent-infant interaction [23], and infantile colic [7].

Since there is no definitive treatment for infantile colic, the mother's inability to help her infant will cause problems in the mother-infant relationship [23]. In some cases, the continuous and non-stop crying of the infant can be a trigger for abuse, neglect and even damage to the infant by the parents and thus endanger the foundation of the family [24, 25].

Several treatment methods are used to treat infant colic; however, for each infant one or a combination of available methods may effectively relieve colic symptoms [26]. Behavioral therapy, counseling support, and parental reassurance are currently considered the most effective therapeutic methods [27]. Today, behavioral counseling specialists believe that a client, who is both the creator and the product of the environment, can guess which behaviors are desirable and then try to transform these guesses and attitudes into actual behaviors [28].

Several similar studies have been conducted in this field, which are somewhat similar to the present study:

Mousavi Asl et al.'s study investigated the effect of relaxation on the health indicators of newborns and it was shown that the colic score of the infant (based on the amount of crying of the infant during the day) decreased with the relaxation of the mother [29]. Keefe et al.'s study, which was based on REST (reassurance, empathy, support, and time-out) intervention at home, showed that the stress level of parents with reassurance counseling, empathy, support, and giving opportunities at home in families that have had restless infants has decreased and communication between parents and infants has improved [30]. Also the study of Makbrian et al., who examined the effect of progressive muscle relaxation along with mental imagery on mental health and mother-fetus attachment during pregnancy, showed that relaxation significantly improved mental health and mother-fetus attachment and has proposed this method as a low-cost and non-pharmaceutical method during pregnancy [31].

Given the prevalence of maternal anxiety in the postpartum period [32], the possible relationship between infantile colic and maternal anxiety [8, 33, 34], negative effects of colic on psychological outcomes and functioning of parents [35], effectiveness of counseling in reducing maternal anxiety [30, 36], and lack of a comprehensive study on the effect of postpartum counseling provided to anxious mothers on infantile colic, this study was conducted to answer the question whether counseling with a behavioral therapy approach in anxious mothers is effective on colic in their infants?

## Methods

### Study design and participants

This randomized clinical trial investigated the effect of counseling with a behavioral approach on infant colic in 46 anxious mothers with infants aged 2 to 6 weeks suffering from colic who referred to the pediatric clinics of Al-Zahra, Taleghani and Children's Hospitals of Tabriz, Iran. The inclusion criteria were mothers of 2–6-weeks-old colicky infants with high PSAS scores (≥ 112) and at least middle school certificates, healthy term exclusively breastfed infants weighed at least 2500 g, gained normal weight, and were diagnosed with infantile colic based on Wessel’s criteria and a pediatrician’s diagnosis. The exclusion criteria included: twins, using any chemical or herbal medicines (*e.g.* anti-flatulence) to treat infantile colic, congenital autoimmune diseases or nutritional problems (the infant), having a history of mental illnesses requiring medication (*e.g.* postpartum depression) in previous deliveries (based on the participant’s statements), mother's anxiety to the extent of needing medication based on her statement, abusing drugs during pregnancy and in the postpartum period (based on the participant’s statements), receiving similar training in the past, attending formal/organized training courses to reduce anxiety in the past, being unsure about their ability to attend all counseling sessions, the occurrence of unfortunate events such as the death of loved ones or divorce, etc. in the past 2 months, and using antibiotics.

The Sample size was determined in G-power based on the study of Aktas et al. [37] It was calculated as 19 by considering the mean infant colic score (m1 = 72.44, SD1 = 9.01), a default 15% decrease in the post-intervention colic score (m2 = 61.574, SD2 = 9.01), α = 0.05, and Power = 95%. The final sample size was then determined as 23 for each group by assuming a loss to follow-up of 20%.

The formula for comparing two means to calculate the sample size was:

$$n=\frac{{\left({z}_{1-\frac{\alpha }{2}}+{z}_{1-\beta }\right)}^{2}\left({s}_{1}^{2}+{s}_{2}^{2}\right)}{{d}^{2}}$$

### Sampling and randomization

The researcher (R.M.) initiated the sampling process after obtaining the approval of Ethics Committee of Tabriz University of Medical Sciences (IR.TBZMED.REC.1398.1083) and registering the study at Iranian Registry of Clinical Trials (IRCT20111219008459N14). Then, she (R.M.) visited the pediatric clinics of Al-Zahra, Taleghani and Children Hospitals of Tabriz and used convenience sampling to select mothers of infants diagnosed with infantile colic (based on Wessel’s criteria and a pediatrician’s diagnosis (M.MG.)).

After providing explanations about the research objectives and methods, mothers who were willing to participate in the study completed PSAS and those with PSAS scores ≥ 112 who also met other inclusion criteria were enrolled after signing informed consent forms. The researcher (R.M) completed the socio-demographic questionnaire, Infant Colic Scale (ICS), and Mother-Infant Attachment Questionnaire (MIAQ) by interviewing the participants.

By considering a 1:1 allocation ratio and using randomized block design (4 and 6-individual blocks), the participants were randomly assigned to the intervention and control groups. The type of each group was written on papers and placed in numbered opaque envelopes in order to conceal the allocation sequence (Sh. H). The participants were then provided with sealed and numbered envelopes based on their enrollment time.

### Intervention

After completing the initial questionnaires (including the socio-demographic questionnaire, PSAS, ICS, and MIAQ), the participants received training about the nature and causes of colic and learned about existing methods used to reduce the severity of colic pain such as massaging, proper breastfeeding, hugging, burping, keeping calm during infant crying, etc*.* As prescribed by a pediatrician, colic drops were used equally for infants of mothers in both groups to control colic pain. Women in the intervention group attended 8 systematic desensitization counseling sessions (2–3 sessions per week) for 4 consecutive weeks in a cozy environment as described below.

- Session 1: Relaxation training for mothers with infants suffering from colic (Teaching proper abdominal breathing).
- Session 2 and 3: Continuation of relaxation training for mothers with infants suffering from colic (Teaching and practicing progressive muscle relaxation technique with proper breathing).
- Session4: Identifying the hierarchy of anxiety of mothers with infants suffering from colic (Listing stressful stimuli of mothers, sequencing and generalizing their mediators, and selecting the main stimulus by mothers).
- Session 5: The beginning of desensitization: the client sitting relaxed or lying down begins to visualize and deal with anxiety-provoking cases (from the least to the most anxiety-causing cases).
- Sessions 6, 7 and 8: Desensitization continues until the person reaches the lowest level of anxiety based on her anxiety hierarchy.

Two weeks after the end of the counseling sessions, the researcher again completed the PSAS, ICS, and MIAQ by interviewing the participants. The control group received routine postpartum training, including breastfeeding and its benefits, self-care after delivery, baby care, etc., and 6 weeks after completing the initial questionnaires, the post-test questionnaires were completed for them too.

### Data collection tools

The data were collected using the socio-demographic questionnaire, PSAS, ICS, and MIAQ. The socio-demographic questionnaire included questions about parents’ age, educational level, and job, family income level, place of residence, and life satisfaction. The content validity of this researcher-made questionnaire was confirmed by 10 faculty members of Tabriz University of Medical Sciences.

#### Infant Colic Scale (ICS)

This 22-item questionnaire was developed by Ellett et al. [38] to assess infantile colic. It includes subscales of cow’s milk/soy protein allergy, immaturity of the gastrointestinal system and CNS, parent-infant interaction, and infant temperament. The items are scored on a six-point Likert scale including strongly agree (score 6), almost agree (score 5), slightly agree (score 4), strongly disagree (score 1), almost disagree (score 2), and slightly disagree (score 3) (Total score range: 22–132). Higher scores indicate greater pain due to increased gas formation. Ellett et al. [38] (2003) confirmed the validity and reliability of this tool. In the present study, a Cronbach’s alpha value of 0.73 was obtained for all items. The psychometric properties of the scale have been confirmed for the Iranian population in a study which is currently under review.

#### Postpartum Specific Anxiety Scale (PSAS)

This 51-item questionnaire measures maternal anxiety using subscales of maternal competence and attachment anxiety, infant safety and welfare anxiety, infant care anxiety, and psychosocial adjustment to motherhood. The items are scored on a four-point Likert scale including highly relevant (score 3), very relevant (score 2), somewhat relevant (score 1), and not relevant (score 0). Streiner et al*. * [39] (2015) calculated the content validity ratio (CVR) of the items to provide a quantitative expression of content validity. The mean CVR for all the items was 0.76 indicating desirable content validity of the whole scale. In addition, the test–retest reliability of 0.88 (*p* < 0.001) revealed excellent stability of PSAS. Hassanzadeh et al*. * [40] standardized this tool in Iran in 2021.

#### Mother-Infant Attachment Questionnaire (MIAQ)

This 19-item scale assessed the attachment of mothers to infants in the 0–36 months-old age group. It is completed by the mother or any person who spends the most time with the infant. The total score ranges from 19 to 95, and higher scores indicate strong mother-infant attachment [41]. In Iran, Hassanpour et al*.* obtained a Cronbach’s alpha of 0.73 for this scale [42].

In this study, the reliability of the questionnaires was assessed using internal consistency (Cronbach’s alpha) and test–retest reliability. The test–retest reliability was assessed by calculating intra-class correlation coefficients (ICC) for 20 mothers who completed the questionnaires twice at a two-week interval. The calculated ICC and Cronbach’s alpha values for ICS, PSAS, and MIAQ included “0.94, 0.91, and 0.93” and “0.70, 0.93, and 0.85”, respectively.

### Statistical analysis

The data were analyzed in SPSS 21. The Kolmogorov–Smirnov (K-S) test results indicated the normality of the quantitative data distribution. The chi-square, Fisher’s exact, and independent t tests were used to examine the homogeneity of the groups in terms of socio-demographic characteristics. The independent t-test and ANCOVA (with controlled baseline values) were used to compare the mean infant colic, postpartum anxiety, and mother-infant attachment scores of the two groups before and two weeks after the intervention, respectively.

## Results

**Fig. 1:**
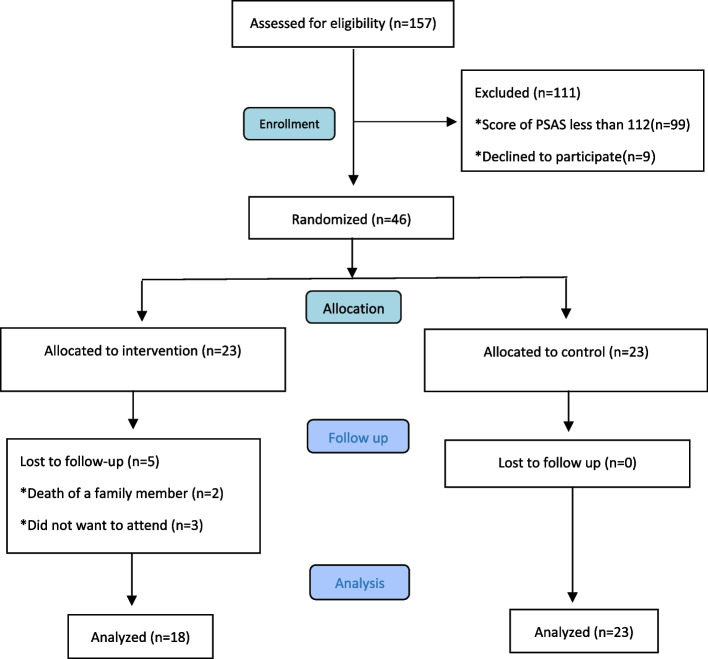
Flow Diagram

This study was conducted between February 2021 and January 2022. The researcher assessed a total of 157 mothers of colicky infants visiting the aforementioned pediatric clinics, of whom 111 individuals were excluded due to low maternal anxiety scores (*n* = 99), unwillingness to participate in the study or uncertainty about participating in all counseling sessions (*n* = 9), and the use of other medicines to treat infantile colic (*n* = 3). In summary, among 157 mothers of colicky infants who completed the PSAS, 58 women had high anxiety scores, and 46 mothers were enrolled as the sample. In addition, 5 mothers withdrew from the intervention group due to the death of their relatives (*n* = 2) and unwillingness to continue participating in the study (*n* = 3). Therefore, the final sample consisted of 41 women (Fig. 1).

As shown in Table 1, there was no significant difference between the intervention and control groups in the socio-demographic profile of participants.

**Table 1:** Socio-demographic characteristics of infants and their parents

	Control group *N* = 23	Intervention group *N* = 18	*p*-value
Age, mother(year)^*^	27.4(5.0)	28.1(3.1)	0/604^**†**^
age, Father (year)^*^	31.5(4.8)	32.9(3.7)	0/269^**†**^
*Gender, infant*			
Female	9(39.1)	11(47.8)	0.552^**††**^
Male	14(60.9)	12(52.2)	
*Number of children*			
1	16(69.6)	13(56.5)	0.542^**§**^
2 or more	7(30.40)	10(43.5)	
*gravida*			
1	13(56.5)	10(43.5)	0.575^**§**^
2 or more	10(43.5)	13(56.5)	
*Number of abortions*			
0	17(73.9)	20(87.0)	0.243^**§**^
1 or more	6(26.1)	3(13.0)	
*Educational level, mother*			0.230^**‡**^
High school	6(26.1)	1(4.3)	
Graduated from high school	6(26.1)	10(43.5)	
college	11(48.7)	12(52.2)	
*Job, mother*			0.608^**§**^
housewife	20(87.0)	22(95.8)	
employed	3(13.0)	1(4.3)	
*Educational level, father*			0.075^**‡**^
High school	2(8.7)	0(0.0)	
Graduated from high school	9(39.1)	6(26.1)	
college	12(52.2)	17(73.9)	
*Job/father*			0.380^**§**^
employee	5(21.7)	5(27.1)	
Manual worker	4(17.4)	1(4.3)	
freelance	14(60.9)	17(74.0)	
*Family income*			0.057^**‡**^
enough	1(4.3)	4(17.4)	
insufficient	7(30.4)	2(8.7)	
Fairly enough	5(65.2)	17(73.9)	
*Habitat*			0.254^**§**^
Private house	3(13.0)	9(39.1)	
Rental house	11(47.8)	7(30.4)	
Mother’s parents home	1(4.3)	0(0.0)	
Father’s parents home	8(34.7)	7(30.4)	
*Life satisfaction*			0.807^**‡**^
satisfied	6(26.1)	6(26.1)	
Fairly Satisfied	14(60.9)	15(65.2)	
dissatisfied	3(13.0)	2(8.7)	

^†^ independent T-test;

^††^chi-square test

^‡^ trend chi-square test

^§^ fisher's exact test;

^*^ mean(standard deviation)

Before the intervention, the independent t-test results showed no significant difference between the mean (standard deviation (SD)) infant colic score in the intervention group (83.1 (13.4)) and that of those in the control group (82.7 (14.8)) (*p* = 0.918). In addition, after the intervention, the results of ANCOVA with controlled baseline values showed no significant difference between the mean (SD) infant colic score of infants of mothers in the intervention group (75.8 (8.9)) and that of those in the control group (79.2 (14.7)) (MD = -2.9, 95% CI = -8.3 to 2.4; *p* = 0.271) (Table 2).

**Table 2:** Comparison of mean infant colic scores before and 2 weeks after intervention between intervention and control groups

Variable	Control group Mean(SD)	Interventiongroup Mean(SD)	Mean Difference (95% CI)	*p*-value
Infant colic score range: 22 to 132				
Before intervention	82.7(14.8)	82.7(14.8)	4.0(8.8 to -9.7)	0.918
Two weeks After intervention	79.2(14.7)	79.2(14.7)	-2.9(-8.3 to 24)	0.271

For comparison of groups before intervention Independent t-test and after intervention, ANCOVA test with controlled baseline values was used

Before intervention, the number of samples in the counseling and control groups was 23, and after the intervention in the counseling and control groups was 18 and 23 respectively

*CI* Confidence Interval, *SD* Standard Deviation

Before the intervention, the number of participants in both the intervention and control group was 23; however, 5 individuals withdrew from the intervention group during the study.

Before the intervention, the independent t-test results showed no significant difference between the mean (SD) maternal anxiety score of women in the intervention group (119.5 (7.3)) and that of those in the control group (120.6 (10.2)) (*p* = 0.694). However, based on the results of ANCOVA with controlled baseline values, after the intervention, the mean (SD) postpartum anxiety score of women in the intervention group (81.8 (41.5)) was significantly lower than that of those in the control group (107.7 (27.3)) (MD = -22.5, 95% CI = -42.7 to -2.3; *p* = 0.029) (Table 3).

**Table 3:** Comparison of mean Postpartum Anxiety scores before and 2 weeks after intervention between intervention and control groups

Variable	Control group Mean(SD)	Interventiongroup Mean(SD)	Mean Difference (95% CI)	*p*-value
Postpartum Anxiety score range: 0 to 153				
Before intervention	120.6(10.2)	119.5(7.3)	-1.0(-6.3 to 4.2)	0.694
Two weeks After intervention	105.7(27.3)	81.8(41.5)	22.5(2.3 to 42.7)	0.029

For comparison of groups before intervention Independent t-test and after intervention, ANCOVA test with controlled baseline values was used

Before intervention, the number of samples in the counseling and control groups was 23, and after the intervention in the counseling and control groups was 18 and 23 respectively

*CI* Confidence Interval, *SD* Standard Deviation

Before the intervention, the independent t-test results showed no significant difference between the mean (SD) mother-infant attachment score of the participants in the intervention group (77.2 (3.6)) and that of those in the control group (77.8 (7.7)) (*p* = 0.763). In addition, after the intervention, the results of ANCOVA with controlled baseline values showed no significant difference between the mean (SD) mother-infant attachment score of members of the intervention group (79.5 (5.7)) and that of those of the control group (79.9 (6.3)) (MD = -0.04, 95% CI = -3.1 to 0.3; *p* = 0.976) (Table 4).

**Table 4:** Comparison of mean Maternal Postnatal Attachment scores before and 2 weeks after intervention between intervention and control groups

Variable	Control group Mean(SD)	Interventiongroup Mean(SD)	Mean Difference (95% CI)	*p*-value
Postnatal Attachment score range: 19 to 95				
Before intervention	77.8(7.7)	77.2(3.6)	-0.5(-4.1 to 3.0)	0.763
Two weeks After intervention	79.9(6.3)	79.5(5.7)	-0.04(-3.1 to 3.0)	0.976

For comparison of groups before intervention Independent t-test and after intervention, ANCOVA test with controlled baseline values was used

Before intervention, the number of samples in the counseling and control groups was 23, and after the intervention in the counseling and control groups was 18 and 23 respectively

*CI* Confidence Interval, *SD* Standard Deviation

## Discussion

Despite the introduction of several treatment approaches for infantile colic, no definitive treatment has so far been provided for this problem and the results of studies conducted in this field are different and inconclusive. Meanwhile, some scholars have recommended the use of parent counseling to inform parents about the nature of colic and the ways of managing it. Moreover, given the possible relationship between infantile colic and maternal anxiety, maternal counseling may reduce mothers’ anxiety and their infants’ colic pain. Therefore this study aimed to determine the effects of behavioral therapy based counseling on infants' colic of anxious mothers.

The results of this study showed that two weeks after the intervention, the mean postpartum anxiety score of mothers in the intervention group was significantly lower than that of those in the control group. The mean infant colic score of the infants of mothers in the intervention group was lower than that of those in the control group two weeks after intervention; but, this difference was not significant between groups. In addition, no significant difference was observed between the mean mother-infant attachment scores, two weeks after the intervention.

Due to the absence of a study completely identical to the present study, studies that are somewhat similar have been discussed:

In this study, counseling sessions provided to the participants reduced their postpartum anxiety. These findings are in line with the following studies: Keefe et al. [30] employed counseling techniques based on the REST (reassurance, empathy, support, and time-out) approach to reduce stress in parents. They showed that the stress level of parents with reassurance counseling, empathy, support, and giving opportunities at home in families that have had irritable infants has decreased and communication between parents and infants has improved. Loghunan et al*. * [43] reduced anxiety levels in mothers by providing CBT-based online counseling sessions. In addition, Mokaberian et al. [31] reduced anxiety levels in women using progressive muscle relaxation with mental imagery technique (a component of behavioral therapy). Therefore, various counseling approaches can be used as cost-effective and non-pharmacological methods to improve emotional stability and enhance mental health of mothers [31, 43].

Counseling sessions decreased the severity of infantile colic in this study; however, this decrease was not statistically significant between groups. This finding is inconsistent with the Results of Mousavi et al. [29] who observed that relaxation exercises performed by mothers significantly decrease their infants’ colic pain scores which are determined based on daytime infant crying. As well, Gordon et al*. * [44] and Salvatore et al*. * [45] found that maternal counseling with reassurance, awareness techniques and nutritional recommendations effectively reduce the duration of infant crying and help mothers prevent functional gastrointestinal disorders in infants. These inconsistencies can be attributed to the use of different infant colic measurement tools and different time periods of interventions (during pregnancy vs postpartum period). A combination of these counseling interventions used to reassure parents and inform them about the nature of colic and existing management methods such as nutritional recommendations, proper breastfeeding, various massages, and pain reduction methods, which was provided to mothers in both groups at the beginning of the present study, seems to help mothers reduce their infants’ colic pain to some extent. However, it seems that all kinds of counseling alone have not been able to have a significant and definitive effect on all infants.

In this study, a high level of mother-infant attachment was observed in the two groups both before and after the intervention. This is considered normal given the importance of family and children in Iranian culture. This result is also in line with the findings of Hassanpour et al*.* in a study carried out in Iran [42]. The study of Dubber et al. [46] about the role of perinatal depression, anxiety and maternal–fetal bonding during pregnancy on postpartum bonding showed moderate maternal anxiety and high mother-infant attachment levels in mothers. They argued that high level of mother-infant attachment may help mothers better control their postpartum anxiety [47]. Mokaberian et al*. * [31] concluded that progressive muscle relaxation with mental imagery technique can be used during pregnancy, as a cost-effective and non-pharmacological method, to improve maternal mental health and enhance mother-fetal attachment. The inconsistency between the result of Mokaberian et al*.* and the present study may be due to different time periods of interventions (during pregnancy vs postpartum period). Moreover, Mokaberian et al*.* examined mother-fetal attachment, while the present study assessed mother-infant attachment.

Although there is no unique treatment for all infants with colic, the present study recommends counseling to control infantile colic along with other colic treatment methods. Maternal counseling can be used as a non-pharmacological and cost-effective method to raise awareness among mothers about the nature of colic and existing management methods. A review of counseling sessions indicates an important part of concerns of Iranian mothers is related to how to take care of the baby, and mothers need a professional companion who can answer their questions and provide them with suitable solutions to various infant problems during pregnancy and the first few months after birth. Therefore it is suggested that in future studies, a health care provider from the time of delivery to at least three months after delivery, support the mother full-time and answer the mother's questions. It is also suggested to conduct similar studies with other counseling approaches on both fathers and mothers.

Supporting women during the postpartum period is one of the most important goals of health care. Today, one of the most important features of health care is solving problems using simple and low-risk methods without drugs. Considering the effect of counseling with a behavioral approach on the anxiety of women with infants suffered from colic in this study, health care workers who have counseling skills can provide the necessary emotional and psychological support for mothers in the postpartum period. Therefore, health care policymakers can play an important role in improving the health of women and their infants by training health care providers in the field of counseling skills.

### Strengths and limitations

To prevent selection bias, the researcher adhered to all principles of clinical trial such as allocation concealment and random allocation. Using the participants’ native language during counseling sessions to better communicate with them was among other strengths of this study. Moreover this was the first study to investigate the effect of behavioral therapy based counseling on infants' colic of anxious mothers.

In the present study, all responses given by participants were assumed to be correct, social desirability in responses and repeated instrumentation (where scores might be higher or lower when participants become familiar with the survey questions) are the other limitations that were beyond the researcher's control. In addition, the participants were all literate, and this can negatively affect the generalizability of the results to illiterate women.

## Conclusion

Behavioral therapy counseling effectively reduced postpartum maternal anxiety; however, the intervention led to an insignificant decrease in the intervention group infants’ colic pain. In addition, no significant difference was observed between the groups in terms of their mean mother-infant attachment scores. Moreover, a high level of mother-infant attachment was observed in the two groups, which is considered normal given the importance of family and children in Iranian culture.

Finally, it can be said that many treatments are being done to control infant colic, which include counseling treatments, but a single treatment that works for all infants is still not available. Therefore, it is possible to benefit from the advice of mothers regarding the knowledge of the nature of colic and the available methods to control it as a non-medicinal and cost-effective method. On the other hand, major part of the worries and anxieties of mothers are caused by not knowing how to take care of an infant especially colicky infants. Therefore, the mother's need for a professional companion who can answer the mother's questions full-time in the first few months after giving birth and provide the mother with suitable solutions for various problems related to taking care of the infant is quite noticeable.

## Data Availability

The datasets generated and/or analyzed during the current study are not publicly available due to limitations of ethical approval involving the patient data and anonymity but are available from the corresponding author on reasonable request.

## Abbreviations

ICS: Infant Colic Scale
PSAS: Postpartum Specific Anxiety Scale
MIAQ: Mother-Infant Attachment Questionnaire
MD: Mean Difference
ICC: Intra Class Correlation

## References

[1] Savino F, Ceratto S, De Marco A, Cordero di Montezemolo L (2014). Looking for new treatments of Infantile Colic. Italian J Pediatr..

[2] Brand S, Furlano R, Sidler M, Schulz J, Holsboer-Trachsler E. Associations between infants’ crying, sleep and cortisol secretion and mother’s sleep and well-being. Neuropsychobiology. 2014;69(1):39–51. 10.1159/000356968 24457194

[3] Milidou I, Sondergaard C, Jensen MS, Olsen J, Henriksen TB (2014). Gestational age, small for gestational age, and infantile colic. Paediatr Perinat Epidemiol.

[4] Wessel MA, Cobb JC, Jackson EB, Harris GS, Detwiler ACJP. Paroxysmal fussing in infancy, sometimes called “colic.” Paediatrics. 1954;14(5):421–35. 13214956

[5] Levitzky S, Cooper R (2000). Infant colic syndrome–maternal fantasies of aggression and infanticide. Clin Pediatr.

[6] Talachian E, Bidari A, Rezaie MH (2008). Incidence and risk factors for infantile colic in Iranian infants. World J Gastroenterol.

[7] Drug and Therapeutics Bulletin Editorial Office LWHJ, UK. Management of infantile colic. 2013;347:f4102. 10.1136/bmj.f4102 23843563

[8] Kaymaz N, Yıldırım Ş, Topaloğlu N, Gencer M, Binnetoğlu FK, Tekin M, et al. Prenatal maternal risk factors for infantile colic. 2015;27(10). 10.7748/ncyp.27.10.32.s28 26654028

[9] Chumpitazi BP, Shulman RJ (2014). Five probiotic drops a day to keep infantile colic away?. JAMA Pediatr.

[10] Saulnier DM, Ringel Y, Heyman MB, Foster JA, Bercik P, Shulman RJ (2013). The intestinal microbiome, probiotics and prebiotics in neurogastroenterology. Gut microbes.

[11] Cryan JF, Dinan TG (2012). Mind-altering microorganisms: the impact of the gut microbiota on brain and behaviour. Nat Rev Neurosci.

[12] Reber SJP. Stress and animal models of inflammatory bowel disease—an update on the role of the hypothalamo–pituitary–adrenal axis. Psychoneuroendocrinology. 2012;37(1):1–19. 10.1016/j.psyneuen.2011.05.014 21741177

[13] Hechler C, Beijers R, Riksen-Walraven JM, de Weerth C (2018). Are cortisol concentrations in human breast milk associated with infant crying?. Dev Psychobiol.

[14] Glynn LM, Davis EP, Schetter CD, Chicz-DeMet A, Hobel CJ, Sandman CAJEHD. Postnatal maternal cortisol levels predict temperament in healthy breastfed infants. Early human development. 2007;83(10):675–81. 10.1016/j.earlhumdev.2007.01.003 17336002

[15] Zijlmans MA, Korpela K, Riksen-Walraven JM, de Vos WM, de Weerth CJP. Maternal prenatal stress is associated with the infant intestinal microbiota. Psychoneuroendocrinology. 2015;53:233–45. 10.1016/j.psyneuen.2015.01.006 25638481

[16] Räihä H, Lehtonen L, Korvenranta HJIMHJ. Family context of infantile colic. Infant Ment Health J. 1995;16(3):206–17.

[17] Papoušek M, von Hofacker NJED, Parenting. Persistent crying and parenting: search for a butterfly in a dynamic system. Early Development and Parenting. 1995;4(4):209–24.

[18] St James‐Roberts I, Conroy S, WilsherKJCc, health, development. Links between maternal care and persistent infant crying in the early months. Child Care Health Dev. 1998;24(5):353–76. 10.1046/j.1365-2214.2002.00089.x 9728283

[19] Seymour M, Giallo R, Cooklin A, Dunning M (2015). Maternal anxiety, risk factors and parenting in the first post-natal year. Child Care Health Dev..

[20] Paulson JF, Dauber S, Leiferman JAJP.Individual and combined effects of postpartum depression in mothers and fathers on parenting behavior. Pediatrics. 2006;118(2):659–68. 10.1542/peds.2005-2948 16882821

[21] Wenzel A, Haugen E, Jackson L, Robinson KJAowsmh. Prevalence of generalized anxiety at eight weeks postpartum. Archives of women's mental health. 2003;6(1):43–9. 10.1007/s00737-002-0154-2 12715263

[22] Telleen SJJoCP. Parental beliefs and help seeking in mothers' use of a community‐based family support program. J Community Psychol. 1990;18(3):264–76.

[23] Hide DW, Guyer BM (1982). Prevalence of infant colic. Arch Dis Child.

[24] Out D, Pieper S, Bakermans-Kranenburg MJ, Zeskind PS, van Ijzendoorn MH (2010). Intended sensitive and harsh caregiving responses to infant crying: the role of cry pitch and perceived urgency in an adult twin sample. Child Abuse Negl.

[25] Barr RG (2002). Changing our understanding of infant colic. Arch Pediatr Adolesc Med.

[26] Ünal ET, Bülbül A, Elitok GK, Avşar H, Uslu S (2021). Evaluation of the Knowledge Level and Attitude of Mothers About Infantile Colic.

[27] Balon AJJAfp. Management of infantile colic. American family physician.1997;55(1):235–42, 45–6. 9012281

[28] M S. Psychotherapy and counseling theories. 1 etnvi. 1379.

[29] Mousavi SA, Ramezani S, Khosravi A (2021). Solution-focused counseling and its use in postpartum depression.

[30] Keefe MR KK, Lobo ML, Kotzer AM, Dudley WN (2006). Reducing parenting stress in families with irritable infants. Nurs Res..

[31] Mokaberian M, Dehghanpouri H, Faez N, Vosadi EJIJoHS. The effect of progressive muscle relaxation with imagery-based relaxation on the mental health and maternal-fetal attachment in women with a first unwanted pregnancy. International Journal of Health Studies. 2021;7(1):11–6.

[32] Akbarzadeh M, Sharif F, Zare N, Ghodrati FJJoFR. Prevalence of symptoms post-partum anxiety and baby blues and factors effective uponit in women with high risk pregnancies. J Fam Res. 2009;5(1):57–71.

[33] Foster JA, Neufeld K-AMJTin. Gut–brain axis: how the microbiome influences anxiety and depression. Trends in neurosciences. 2013;36(5):305–12. 10.1016/j.tins.2013.01.005 23384445

[34] Carey WBJCP. Maternal anxiety and infantile colic: Is there a relationship? Clinical Pediatrics. 1968;7(10):590–5. 10.1177/000992286800701007 5682375

[35] Abacı FB, Gökçe S, Tuygun N, Karacan CD, Öner ÖJTJP. Psychosocial status and quality of life in mothers of infants with colic. Turk J Pediatr. 2013;55(4):391–5. 24292032

[36] Green SM, Donegan E, McCabe RE, Streiner DL, Agako A, Frey BNJA, et al. Cognitive behavioral therapy for perinatal anxiety: a randomized controlled trial. Aust N Z J Psychiatry. 2020;54(4):423–32. 10.1177/0004867419898528 31957479

[37] Aktas S, Kucuk Alemdar D. Correlation between Infantile Colic and Maternal Breastfeeding Self-Efficacy, Breastfeeding Success and Breast Milk Amount. J Tropical Pediatr. 2019;65(4):321–7. 10.1093/tropej/fmy054 30137617

[38] Ellett MLC, Murphy D, Stroud L, Shelton RA, Sullivan A, Ellett SG, et al. Development and psychometric testing of the infant colic scale. Gastrointest Nurs. 2003;26(3):96-103. 10.1097/00001610-200305000-00002 12811319

[39] Streiner DL, Norman GR, Cairney J (2015). Health measurement scales: a practical guide to their development and use.

[40] Hasanzadeh R, Jafarabadi MA, Hasanpour S, Fallon V, Silverio SA, Montazeri R, et al. Psychometric evaluation of the postpartum specific anxiety scale in an Iranian population (PSAS-IR). BMC Pregnancy and Childbirth. 2021;21(1):1–7. 10.1186/s12884-021-04085-w PMC8417620 34481468

[41] Condon JT, CorkindaleCJJJoR, Psychology I. The assessment of parent-to-infant attachment: development of a self-report questionnaire instrument. J Reprod Infant Psychol. 1998;16(1):57–76.

[42] Hasanpour S, Ouladsahebmadarek E, Hosseini MB, Mirghafourvand M, Heidarabadi S, Jafarabadi MAJd. Mother-infant attachment at the age of 1 year in recipients of developmental care after preterm birth. Int J Women's Health Reprod Sci. 2018;21:22.

[43] Loughnan SA, Butler C, Sie AA, Grierson AB, Chen AZ, Hobbs MJ (2019). A randomised controlled trial of ‘MUMentum postnatal’: internet-delivered cognitive behavioural therapy for anxiety and depression in postpartum women.

[44] Gordon M, Gohil J, Banks SS. Parent training programmes for managing infantile colic. Cochrane Database of Systematic Reviews. 2019(12). 10.1002/14651858.CD012459.pub2 PMC6890412 31794639

[45] Salvatore S, Abkari A, Cai W, Catto‐Smith A, Cruchet S, Gottrand F, et al. Review shows that parental reassurance and nutritional advice help to optimise the management of functional gastrointestinal disorders in infants. Acta paediatrica. 2018;107(9):1512–20. 10.1111/apa.14378 PMC6120453 29710375

[46] Dubber S, Reck C, Müller M, Gawlik SJAowsmh. Postpartum bonding: the role of perinatal depression, anxiety and maternal–fetal bonding during pregnancy. Archives of women's mental health. 2015;18(2):187–95. 10.1007/s00737-014-0445-4 25088531

[47] Duran S, Kaynak SJEJFM. Investigation of the Relationship Between Postpartum-Specific Anxiety and Maternal Attachment and Affecting Factors in a Turkish Sample. Euras J Fam Med. 2021;10(4):219–26.

